# Editorial: Mechanisms of abiotic stress responses and tolerance in plants: physiological, biochemical and molecular interventions, volume II

**DOI:** 10.3389/fpls.2023.1272255

**Published:** 2023-09-13

**Authors:** Shabir Hussain Wani

**Affiliations:** Mountain Research Centre for Field Crops, Khudwani, Sher-e-Kashmir University of Agricultural Sciences and Technology, Srinagar, Jammu and Kashmir, India

**Keywords:** abiotic stress, molecular mechanism, drought, phytohormomes, salinity, transcription factors

Amid climate change, abiotic stresses such as extreme temperatures, drought, salinity, and heavy metal toxicity significantly impact plant growth and productivity, leading to impaired morphological development and negative effects on plant health (Hasanuzzaman and Fujita, 2022; Bhardwaj et al., 2023). These stresses cause morphological changes in plants, such as reduced shoot and root growth, poor anther dehiscence, loss of pollen viability, increased flower drop, decreased flower fertilization, seed shrinking, and shortened grain filling periods. In addition, leaf senescence, chlorosis, necrosis, burning, and abscission further contribute to the detrimental impacts on plant growth. (Saxena et al., 2019; Dumanović et al., 2021; Hasanuzzaman and Fujita, 2022; More et al., 2023). To counteract these detrimental effects, plants employ various adaptation and tolerance mechanisms. Recent studies have focused on unraveling the mechanisms underlying plant responses to abiotic stress. Physiological interventions, such as stomatal regulation mediated by the abscisic acid (ABA) signaling pathway, ion homeostasis, and osmotic adjustment, are crucial for plant adaptation to drought and salt stress (Kuromori et al., 2022; Li et al., 2020). Additionally, the involvement of reactive oxygen species (ROS)-scavenging enzymes and antioxidant systems in mitigating heat-induced oxidative damage and promoting thermotolerance has been elucidated (Dumanović et al., 2021; Mittler et al., 2022). The crosstalk between hormonal signaling pathways and the regulation of antioxidant defence systems, ion homeostasis, and osmotic adjustment have been highlighted (Ramegowda et al., 2020; Singhal et al., 2021). Genome-wide transcriptomic studies have provided valuable insights into stress-responsive genes including transcription factors, microRNAs, and stress-responsive proteins (Liu et al., 2022). The successful application of CRISPR-Cas9 technology has been observed in developing abiotic stress-resilient crops, facilitated by bioinformatics tools for designing suitable CRISPR/Cas9 vectors (Zafar et al., 2020; Wang et al., 2022). Understanding the mechanisms underlying abiotic stresses is crucial for the development of strategies that enhance plant stress tolerance and ensure sustainable agriculture. By uncovering the roles of physiological, biochemical, and molecular interventions, researchers aim to promote the resilience of crops to abiotic stresses, thus safeguarding global food security in the face of abrupt global climate change.

In this Research Topic, 35 articles have been put together, which comprise of original research as well as review articles, to obtain the crux of the variety of intrusions made by global researchers through application of advances in physiological, biochemical and molecular interventions to better understand the mechanisms underlying abiotic stress responses and tolerance in plants.

To advance a context on the theme and to lay a sturdy footing for the Research Topic, nine review articles have been published. One, entitled *“The multifaceted roles of MYC2 in plants: Toward transcriptional reprogramming and stress tolerance by Jasmonate signaling*” by Song et al., details recent investigations in several facets of *MYC2*-mediated JA signaling and their role in plant growth and stress response. Melatonin controls plant stress responses by unswervingly impeding the deposition of reactive oxygen and nitrogen species, and by circuitously affecting stress response pathways. Therefore, a review on “*Melatonin-mediated abiotic stress tolerance in plants*” by Zeng et al. was also included in this Research Topic. In this review, a recent update on melatonin biosynthesis, metabolism, and antioxidation was highlighted, and further emphasis was given to melatonin-mediated tolerance to abiotic stresses including drought, waterlogging, salt, heat, cold, heavy metal toxicity, and light-induced stresses. It was also proven that melatonin acts as a signaling molecule, regulating the expression of genes involved in stress responses, antioxidant production, and phytohormone pathways, such as ABA, ET, and JA. In a recent study by Amin et al., the exogenous application of melatonin resulted in improved low temperature and high humidity stress tolerance in cucumber. It also resulted in an upsurge in the transcript levels of genes encoding antioxidant enzymes under stress conditions. Another review entitled “*Phytohormones trigger drought tolerance in crop plants: Outlook and future perspectives*” by Iqbal et al. emphasized the role of phytohormones in drought tolerance via regulation of morphological, physiological, biochemical, and molecular processes. The morphological and physiological processes encompass vagaries in the composition of leaves, root growth, and stomatal control. The biochemical method consists of altering the levels of phytohormones. Molecular progressions comprise phytohormone-mediated signals, leading to the stimulation of many transcription factors that ground the expression of genes essential for plant endurance under abiotic stresses, particularly drought stress. Improving the water use efficiency (WUE) in winter wheat using agronomic practices has been proposed by Adil et al., which is described in a systematic review entitled “*Effects of fallow management practices on soil water, crop yield and water use efficiency in winter wheat monoculture system: A meta-analysis*”. In this review, the authors conclude that no tillage in combination with straw mulching significantly increased WUE and wheat yield under erratic climatic conditions.

Further, another review entitled “*Recent advancement in OMICS approaches to enhance abiotic stress tolerance in legumes”* by Ali et al. described the role played by genomics, transcriptomics, metabolomics, and proteomics for elucidating the complex abiotic stress mechanisms in legumes. The authors also gave an idea about the application of genome editing and genomic selection for abiotic stress mitigation in legumes and concluded that there is a need to change the narrative regarding orphan crops for legumes, as nowadays most legumes have been studied deeply and are at par with cereal crops in terms of genomics resources available in databases. Another review entitled “*Advances in “Omics” approaches for improving toxic metals/metalloids tolerance in plants”* by Raza et al. focused on the current novelties in “omics” that possibly accelerate the progress of toxic metals/metalloids tolerant plants.

One imperative area in the field of abiotic stress mitigation is engineering of the plant microbe interaction; therefore, a review entitled *“New opportunities in plant microbiome engineering for increasing agricultural sustainability under stressful conditions”* by Afridi et al. was also included. This review resolved various facts, for instance, the effect of secondary metabolites of microorganisms on beneficial microbes of the plant microbiome, variation of constant environmental condition and their influence on the host and its allied microbial communities, and the amalgamation of agronomic practices with synthetic biology and their adjustment and suitability to each other. There is enough evidence that microbes play a role in plant drought tolerance, which obliged us to include another review entitled *“Research progress in the field of microbial mitigation of drought stress in plants”* by Shaffique et al., in which stress was waylaid upon instigation of drought tolerance arbitrated by plant inoculation with fungi, bacteria, viruses, and quite a few bacterial elements. It was concluded that microbial connotations play a probable role in facilitating plant protection responses to drought, which is a vital aspect for agricultural production systems that are affected by climatic aberrations.

Silicon amends biotic and abiotic stress situations in plants by regulating the physiological, biochemical, and molecular responses. Therefore, another review on the *“Multidimensional role of silicon to activate resilient plant growth and to mitigate abiotic stress*” by Mir et al. was included in this Research Topic. All of these reviews helped to further sharpen the concepts and processes related to advancements in the field of abiotic stress, such as drought and toxic metalloid stress mitigation using state of the art biochemical, physiological, and molecular approaches in various crop plants.

Salinity is one of the most damaging abiotic stresses affecting 7% of land area and 33% of irrigated lands worldwide. Extricating the specific functions of various stress-related genes, including those responsible for salinity stress in plants, is still a challenge in many crops, and such gene products have been categorized as hypothetical domains of unknown function, or *DUFs*. These are characterized in the *Pfam* database and named using the prefix *DUF* followed by a number, for example, *DUF1* and *DUF2*. These proteins are extensively distributed in different plants and restrain at least one extremely conserved domain of *DUF* (Bateman et al., 2010). Zaynab et al. reported that genome-wide identification and expression profiling of the *DUF221* gene family offers innovative understandings into abiotic stress, including salt stress responses in potato. The authors also concluded that *StDDP7* exhibited high transcript abundance against salt stress in potatoes. Germin-like proteins (*GLPs*) are persistent proteins considered to be water-soluble glycoproteins, and may be involved in different abiotic and biotic stresses (He et al., 2021). To decipher the possible involvement of *GLPs* in potato, a comprehensive genome-wide analysis was executed in the potato genome by Zaynab et al., which led to identification of 70 *GLPs*. Gene structure, synteny, phylogenetic motifs, promotor, and miRNA analyses were carried out to further elucidate the mechanisms involved. Further, the *StGLP5* gene exhibited the highest expression in response to salt stress. Plant growth-promoting rhizobacteria (PGPR) are the most auspicious advantageous microorganisms that can be used to progress plant responses against biotic and abiotic stresses, including salinity stress. Ali et al. demonstrated that *Bacillus thuringiensis* PM25 ameliorates oxidative damage caused by salinity stress in maize by regulating growth, leaf pigments, the antioxidant defence system, and stress responsive gene expression. This study also revealed that *B. thuringiensis PM25* plays an imperative role in reducing salinity stress by regulating antioxidant defence and abiotic stress-related genes.

To counter the deleterious effects of salinity stress, plants adopt physio-biochemical and molecular signaling defensive mechanistic cascades to prevail over salinity stress; however, unrelenting vulnerability can overcome the defence system, causing cell death and the breakdown of crucial gadgets. Taming plant vigour and defence responses can thus escalate plant stress tolerance and productivity. On the other hand, the apparently vital element silicon (Si)—the second-most widely available element in the Earth’s crust—is employed by plants and applied exogenously to fight salinity stress and progress plant growth by augmenting physiological, metabolomic, and molecular responses (Khan et al., 2019). To support the above fact, Murad and Muneer studied the beneficial effect of silicon supplementation, which resulted in increased growth in mungbean and alleviated salinity stress as evidenced by the results of their physio-chemical experiments. Similarly, in another study by Adhikari et al., silicon applied in combination with biochar, K_2_HPO_4_, and rhizospheric fungus *Curvularia lunata* isolate (AR11) significantly improved the salt and drought stress tolerance in rice. Such studies help to lower fertilizer use, production costs, and environmental pollution, minimize food toxicity, and foster sustainable agriculture.

A foliar spray of antioxidants is a cost effective and realistic tactic to fight the various deleterious effects of salinity stress in agricultural crops. Naqve et al. demonstrated that foliar application of α-tocopherol reduced salinity-induced impairment in okra by enhancing growth, photosynthetic pigments, and leaf gas exchange traits, and by conceivably shielding chloroplasts as a result of its antioxidant potential. Foliar application of H_2_O_2_ led to an increase in plant growth of *Ficus deltoidea* under drought stress via accumulation of metabolites and Rubisco gene expression (Khandaker et al.). The authors further demonstrated that application of H_2_O_2_ resulted in elevated photosynthesis, stomatal conductance, chlorophyll fluorescence, carotene, total phenols, total flavonoids, sugar content, and antioxidant activities under drought stress conditions. Defensin genes are included in a plant’s defence system and are triggered when plants are subjected to biotic or abiotic stress. They play a key role in regulating several signaling pathways responsible for many plant defence systems. Jabeen et al. reported the isolation and characterization of the *peroxidase P7-like* and *Rab-GDI* like genes, which are novel defensin genes from particular medicinally important plants, to explore their signaling mechanisms and defence-associated roles for breeding.

Heavy metal (HM)-arbitrated poisonous effects on plants have received substantial attention worldwide as they unswervingly lurk in the food supply chain. Plants growing in Cd-polluted soils constitute cadmium’s main entry into the food chain, presenting a serious threat to animal and human health (Peralta-Videa et al., 2009). A study on the extrinsic application of salicylic acid and hydrogen peroxide to ameliorate cadmium stress in milk thistle was carried out by Nizar et al., which revealed that the toxic effects of heavy metal (Cd^2+^) impeded the morphological and physiological traits of milk thistle. However, priming and foliar treatment of plant-signaling molecules with SA and H_2_O_2_ exhibited a constructive influence on decreasing the toxic effect of Cd^2+^. The secondary metabolites displayed enhanced tolerance to the Cd^2+^ concentration. Their work suggests mining of the genes responsible for amending the heavy metal toxicity in medicinal plants of economic importance grown at varied altitudes, for which very few studies have been conducted. Another article by Kanu et al. described the detrimental effects of Cd stress on fragrant rice cultivar; exogenous application of methyl jasmonate resulted in improved antioxidant activity and chlorophyll content and reduced oxidative damage under Cd stress conditions. According to Zaid et al., the application of plant growth regulators such as salicylic acid helps to lower Cd uptake, mitigates Cd toxicity, and improves the yield and quality traits, thus serving as a remunerative, concrete, and viable tactic for crop plants in general and for medicinal and aromatic plants in particular. A similar study by Ilyas et al. showed that *Pennisetum purpureum* and *Paspalum dilatatum* are bio-accumulators of cadmium and can be suggested for plantation in Cd-contaminated soils. Further, probable incursion by alien plants in contaminated soil environments takes place within the introduced array. Therefore, non-invasive alien plants and native plants should be endorsed to enable land phytoremediation in contaminated environments. Another study tackling the detrimental effects of the combined stress of Cd and dichlorodiphenyltrichloroethane (DDT) was conducted by Mubeen et al., in which a novel approach using calcium nanoparticles impregnated with benzenedicarboxylic acid was used to alleviate the combined stress of DDT and cadmium in Kale. The growth and physiological characteristics of Kale were efficaciously enhanced by Ca- and Bd-dependent Bd_Ca_ under several stress conditions caused by Cd and DDT pollution. By reducing the biosynthesis of ROS, improving the antioxidative machinery, and modulating the synthesis of osmoregulators, the externally applied Bd_Ca_ augmented plant resistance to the combined abiotic stressors. In addition, Bd_Ca_ repressed the accumulation of DDT in plant tissues and reduced its translocation factor. Consequently, innovative treatment with Bd_Ca_ is suggested for mitigating manifold stresses to ensure a viable crop production system. Compatible solute accumulation, such as proline, in plants in response to abiotic stress is a well-known phenomenon. To check the preventive property of accumulated proline against Cd stress, a study was conducted by Garcia de la Torre et al. on transgenic and wild type *Medicago trunculata* plants. The results reported in this study were in line with the earlier proven fact that the expression of *VaPC5S* and the ensuing high proline accumulation resulted in alleviated Cd stress in *M. truncatula* transgenic plants. Conversely, this upsurge in tolerance is not exclusively due to proline build-up, but to a proline-induced upregulation of many vital genes associated with proline uptake, phyto-chelatins biogenesis, antioxidant machinery, and *NADPH*-recycling in the transgenic plants, which turn out to be well-armoured to tackle the Cd stress. Similarly, Shah et al. reported that *Indole pyruvate decarboxylase gene (IDPC)* regulates the auxin synthesis pathway in rice by interacting with the in*dole-3-acetic acid–amido synthetase* gene, promoting root hair development under cadmium stress. *IPDC* transgenic lines exhibited a 40% surge in lateral roots. The results showed an increase in root hairs (RHs) and lateral root density, as well as changes in auxin levels and the expression of the *YUCCA* gene.

A considerable increase in the atmospheric CO_2_ level has occurred since the industrial revolution, and it is anticipated that it will reach 700 ppm by the year 2050 (IPCC, 2018). This upsurge in atmospheric CO_2_ will result in global warming (Mikhaylov et al., 2020), eventually leading to water stress, a major limiting factor of crop productivity. However, increased CO_2_ (eCO_2_) is thought to hasten the photosynthetic rate and hinder photorespiration (Li et al., 2019). This fact was supported by results from Javaid et al., wherein the authors studied the photosynthetic response and biomass productivity in *A. longifolia* under elevated CO_2_ and water limited conditions. eCO_2_ enhanced the photosynthetic activity, water use efficiency, and general growth-related traits of *A. longifolia ssp. longifolia* under both well-watered and water limited conditions.

The spatial architecture of a root system has a key influence on plant growth and development, which includes the firmness of the soil, uptake of water and essential nutrients, and interrelation with beneficial soil microorganisms. Root system architecture is quantified by numerous parameters. Another vital architectural factor is the alignment of root growth (Waite et al., 2020). Therefore, interest has grown among scientists working on plant stress biology. As a consequence, the gene *DEEPER ROOTING 1* (*DRO1*) was first mined in rice from a quantitative trait locus associated with root orientation and overall root system depth (Uga et al., 2013). Recently, ABA inducible *DRO1* was found to improve the adaptation to water stress in maize (Feng et al., 2022). Sun et al. studied the expression of Potato *StDRO1* in *Arabidopsis* in terms of DRO1 function for the control of root architecture and drought tolerance. The ectopic expression of *StDRO1* in *Arabidopsis* generated a substantial upsurge in biochemical parameters (e.g., *SOD*, *POD*, and *CAT*) and proline content under drought stress conditions, which showed that *StDRO1* is likely a major player for potato drought stress tolerance. The above, and similar related studies published in the literature support the hypothesis of improving root architecture for abiotic stress tolerance in plants. In a recent study on passion fruit by Rizwan et al., important transcription factors (TFs) such as *ERF*, *AP2*, *MYB*, and *bZIP* were predicted and envisaged in a TF regulatory network with *PeCER* genes. Gene ontology (GO) and Kyoto encyclopaedia of genes and genomes (KEGG) annotation analyses showed that *PeCER* genes were particularly associated with fatty acid, cutin, wax biosynthesis, plant-pathogen interactions, and stress response pathways. It has been shown in several studies that drought tolerance is regulated by many genes, including TFs, that permit plants to endure adverse conditions. These remain likely genomic candidates for extensive application in crop breeding. These TFs consist of vital molecular switches managing the control of plant developmental processes in response to an array of stresses (Joshi et al., 2016). Wang et al. reported that *NAC* Transcription Factor *TwNAC01* positively regulates drought stress responses in *Arabidopsis* and *Triticale*. Overexpression of the transcription factor gene *TwNAC01* in *A. thaliana* improved the drought tolerance of *A. thaliana* by boosting the water holding tendency of the leaves, lowering cellular membrane injury, reducing the production of ROS in the leaves, and promoting root elongation. Plant growth and development is impaired due to growing abiotic and biotic stresses. To counter these stress conditions, the majority of the plants are shielded with a hydrophobic protective layer generally known as cuticle wax, which is the primary blockade between the environment and plants (Trivedi et al., 2019). Cuticle wax comprises very-long-chain fatty acids (*VLCFAs*) and their cognates. β-Ketoacyl-CoA synthase (*KCS*) is a crucial enzyme in the development of *VLCFAs* and offers a precursor for the synthesis of cuticle wax. Rizwan et al. reported the identification of 32 *KCS* genes in the passion fruit genome, and phylogenetically grouped them as *KCS1*-like, *FAE1*-like, *FDH*-like, and *CER6*-like. Moreover, 31 *PeKCS* genes were positioned on seven chromosomes, while one *PeKCS* was localized to the unassembled genomic scaffold. Cis-element analysis allows understanding of the likely role of *PeKCS* genes in phytohormones and stress responses.

In another interesting measure to tackle abiotic stress such as heat stress, Kareem et al. demonstrated that zinc oxide nanoparticles interact with physiological and biochemical traits in terminal heat stress mitigation in mungbean (*Vigna radiata* L.). Nanotechnology is transforming global agriculture, particularly zinc oxide nanoparticles (nano-ZnO), which have garnered substantial research interest due to their unique properties and countless practical applications against abiotic stresses. Thus, the foliar application of nano-ZnO can be recommended as a robust way to shield crops from heat stress-mediated impairment, with low chances of nanoparticle release to the environment.

Sunlight releases extensive radiation such as visible light, ultraviolet (UV), and infrared radiation that are finally received by the surface of the earth (Kumar et al., 2016). Among these different types of radiation, UV rays affect the development of plants during their life cycle. UV-B radiation strength is largely affected by the depth of the stratospheric ozone layer and is particularly damaging to plants (Sztatelman et al., 2015). Sah et al. reported the proteomic responses of rice (*Oryza sativa*) leaves to UV-B radiation stress. Protein expression under various levels of UV-B was observed in rice cultivars, and it differed among the two rice varieties under study. The differentially expressed proteins reported in the above study are related to plant growth and development, cell defence and redox homeostasis, metabolism, cell wall architecture, photosynthesis, signal transduction, stress response, and ABA signaling. In another study, Sidibe et al. concluded that transcriptome analysis of lettuce in response to UV-C alone or together with Xcv revealed that the differentially expressed genes (*DEGs*) are linked to homeostasis, growth, and defence. To wrap up, UV-C hormesis applied in the present study is a potent eustress that does not hinder the capability of treated plants to restart normal growth or to guard themselves against possible stressors.

The detrimental effect of the herbicide atrazine in the form of its persistence for a long duration in soil has been reported. Sun et al. integrated metabolomics and transcriptomics for investigating the tolerance of Foxtail millet (*Setaria italica*) to atrazine stress. The results indicated that 2,208 *DEGs* and 192 differentially expressed metabolites (*DEMs*) were recognized in atrazine-treated GA2. A bioinformatics analysis of *DEGs* and *DEMs* revealed that several biosynthetic metabolism pathways were suggested to be augmented in GA2, such as glutathione metabolism, amino acid biosynthesis, and phenylpropanoid biosynthesis. In this study, an atrazine-resistant millet variety was investigated, which resulted in the generation of valuable data that may help refine our understanding of the multifaceted molecular apparatus underlying the response to atrazine stress in millet.

Contemporary studies including full genome sequencing, mutational transgenic plant analyses, and genome editing have facilitated a profound perception of the multifaceted transcriptional machinery that operates under cold stress in plants. Variations in gene expression in response to cold stress are followed by upsurges in the levels of numerous metabolites with defending effects to counter the detrimental effects of cold stress (Sanghera et al., 2011; Islam et al., 2023). Among the various gene types whose role in cold stress has been elucidated, compatible solutes, including trehalose, may play a major protective role. In a recent study, Raza et al. demonstrated that the exogenous application of trehalose augmented the osmoprotectants soluble sugar (*SS*), soluble protein (*SP*), and proline (Pro), as well as the activities of antioxidant enzymes such as catalase (*CAT*), peroxidase (*POD*), superoxide dismutase (*SOD*), and ascorbate peroxidase (*APX*) under cold stress conditions. Therefore, trehalose controls the antioxidant and osmotic balance and also plays an active role in metabolism and the signaling network to improve the cold stress tolerance in crops such as rapeseed.

In conclusion, abiotic stresses still pose a great threat to sustainable agriculture nowadays and in the near future, and with the advent of erratic weather conditions and extreme temperatures as a consequence of climate change, these abiotic stresses will become more lethal for crop growth and productivity. Adequate research has been conducted to unravel the physiological, biochemical, and molecular mechanisms underlying abiotic stress tolerance in crop plants. More focused research needs to be carried out to better comprehend plant responses to abiotic stresses. This is conceivable with the dawn of emerging scientific inventions in the field of plant biology, such as genome editing and the advancement in artificial intelligence, which aid the food requirements of the ever-increasing human population.

## Author contributions

SW: Writing – original draft, Writing – review & editing.
